# Understanding seasonal migration of Shishamo smelt in coastal regions using environmental DNA

**DOI:** 10.1371/journal.pone.0239912

**Published:** 2020-10-01

**Authors:** Tetsu Yatsuyanagi, Hitoshi Araki

**Affiliations:** 1 Graduate School of Agriculture, Hokkaido University, Sapporo, Hokkaido, Japan; 2 Research Faculty of Agriculture, Hokkaido University, Sapporo, Hokkaido, Japan; University of Hyogo, JAPAN

## Abstract

Migratory organisms have their own life histories that efficiently link multiple ecosystems. Therefore, comprehensive understanding of migration ecologies of these organisms is essential for both species conservation and ecosystem management. However, monitoring migration at fine spatiotemporal scales, especially in open marine systems, often requires huge costs and effort. Recently, environmental DNA (eDNA) techniques that utilize DNA released from living organisms into their environment became available for monitoring wild animals without direct handling. In this study, we conducted an eDNA survey for understanding marine migration of an endemic fish species, Shishamo smelt (*Spirinchus lanceolatus*). We examined 1) seasonal habitat changes in coastal regions and 2) environmental factors potentially driving the migration of this species. The eDNA concentrations along a 100 km-long coastline exhibited spatiotemporal variation, suggesting that this species shifts their habitat away from nearshore areas between spring and summer. We also found a significantly negative association between the eDNA concentration and sea surface temperature. That finding suggests that the offshore migration of this species is associated with increased sea surface temperature. This study reveals new aspects of *S*. *lanceolatus* life history in coastal regions. Together with our previous eDNA study on the freshwater migration of *S*. *lanceolatus*, this study illustrates the potential of eDNA techniques for understanding the whole life history of this migratory species.

## Introduction

Migratory organisms move across heterogeneous ecosystems. Their life-history strategies not only determine the fate of individuals but also influence structures and dynamics of populations, communities, and ecosystems [1]. For example, anadromous fishes migrate between saltwater and freshwater to complete their life cycles. These large-scale migrations sustain ecosystem structures and functions through food webs and by transporting marine-derived nutrients to riparian zones [2–4]. Because such migrations can be the key to maintaining community dynamics and ecosystem functions, proper monitoring of fish migration is essential for both species conservation and ecosystem management.

Shishamo smelt (*Spirinchus lanceolatus*), in the family Osmeridae, is an anadromous forage fish endemic to the Pacific regions of Hokkaido, Japan [5]. This forage fish is expected to contribute to energy supplies for seabirds, marine mammals, terrestrial animals, and predatory fish species. *S*. *lanceolatus* also has a significant economic value for commercial fisheries and is known as a local specialty product, despite the species listing as threatened on the Japanese Red List due to its limited distribution and resource degradation [6]. In early winter, *S*. *lanceolatus* migrates into rivers for reproduction at one or two years of age. Previous studies investigated their reproductive migration by examining the spatial distribution and environmental conditions of the spawning grounds [7] and temporal migration dynamics [8]. However, two important questions about marine migration of this species are unsolved to date: (i) how do *S*. *lanceolatus* move through seawater habitats? (ii) What environmental factors (if any) trigger their habitat changes? While commercial fisheries target this species in coastal regions around October, distribution trends through the other seasons are almost entirely unknown.

Investigating migration at fine spatial and temporal scales is often very challenging due to huge effort and cost requirements, especially in open marine systems (e.g., catch-based survey on a research vessel). While bio-mechanical methods such as biologging, biotelemetry, or GPS techniques may enabled us to track species’ movements in detail [9, 10], the recovery rates of tracking instruments are often very low, and these surveys require certain fish sizes and durability to fit those instruments. Moreover, in the case of migration ecology studies, it is often necessary to visualize movement at the population level; therefore, individual movement data often have limited use [11].

Environmental DNA (eDNA) analysis, to trace organismal DNA found in the environment, can be an alternative way to study fish migration [12]. By detecting and quantifying eDNA, researchers can estimate the presence, absence, and abundance of target species’ populations in aquatic systems [13–17]. Detections of eDNA may also provide useful information about seasonal movements or habitat changes of target species in various environments, such as rivers [18–20], lakes [21, 22], and estuaries [23]. Even in marine environments, eDNA surveys have displayed their abilities to estimate spatial abundance and biomass [24–26], and the community composition of various species [27–32]. These labor-saving sampling processes enable us to carry out large-scale surveys in fine spatiotemporal scales and can be applied to quantitative analysis of species migration.

In this study, we conducted an eDNA survey from March to August along the Pacific coast of Hokkaido to gain an understanding of the seasonal habitat changes of *S*. *lanceolatus* at sea. Firstly, we investigated spatial and temporal eDNA concentrations at fixed sampling points nearshore using the species-specific eDNA detection system established in our previous study [8]. Next, we evaluated the relationships between eDNA concentrations and environmental factors. Since the shallow coastal environment has unique properties such as strong seasonal variation in temperature [33] and high productivity by phytoplankton [34], we tested a hypothesis that one or a few of these environmental factors are associated with the seasonal habitat changes of *S*. *lanceolatus* in coastal regions.

## Materials & methods

### Ethical statement

We declare no ethical statement. There was no need to obtain any permission for conducting this study including field samplings and lab experiments because water sample collection in our study sites is not prohibited, and no animals were captured or killed in our experiments.

### eDNA sample collection

**Fig 1:**
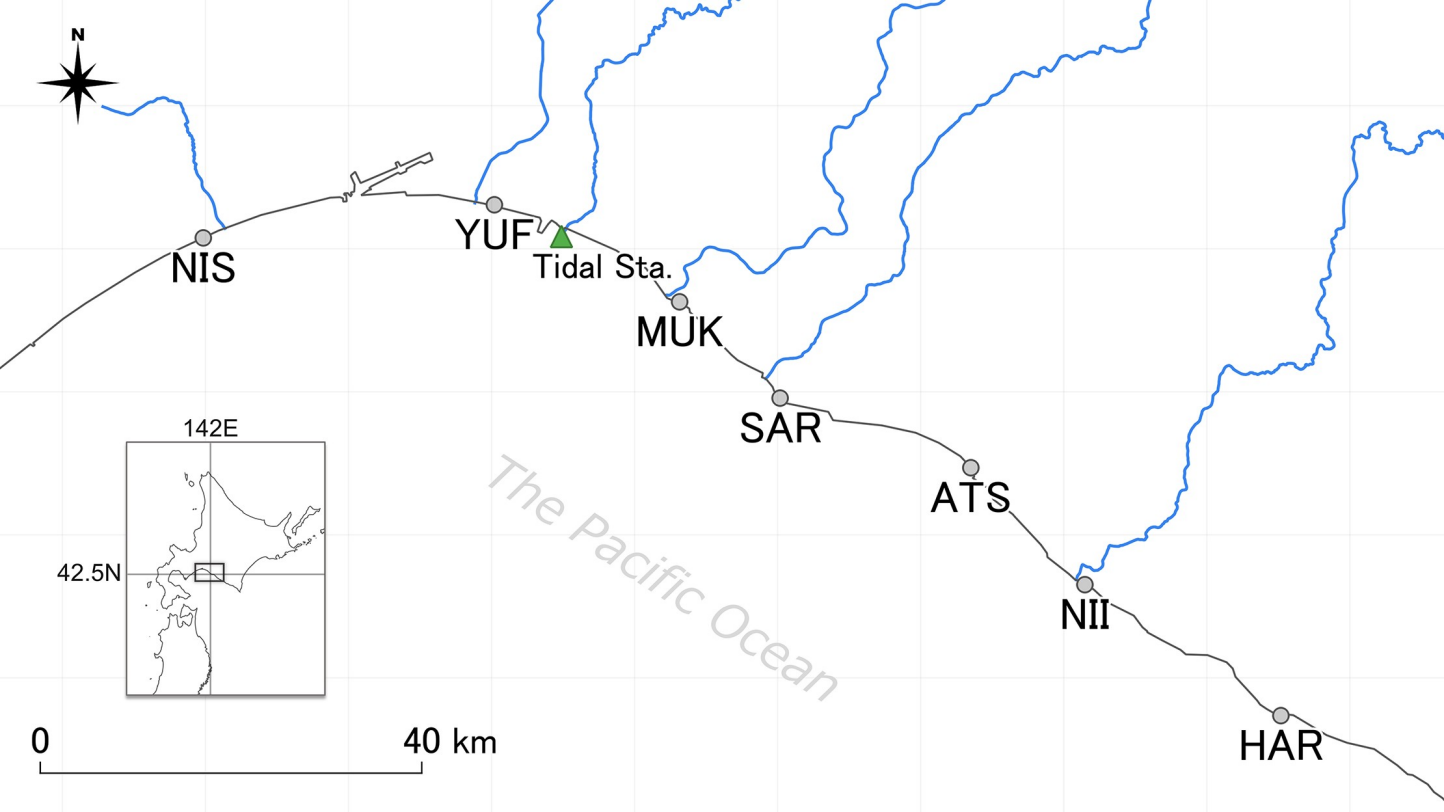
Locations of eDNA sampling sites. Seven gray dots show the site locations with capitalized abbreviations of each name. A green triangle named “Tide Sta.” indicates the location of the tidal observation station. Blue lines illustrate rivers that *S*. *lanceolatus* is believed to migrate into. The river and coastline data were provided by the National Land Information Division, Ministry of Land, Infrastructure, Transport and Tourism of Japan, under the CC BY 4.0 license.

Seven locations along the western Pacific coast of Hokkaido were selected as eDNA sampling sites (Nishikioka, Yufutsu, Mukawa, Saru, Atsuga, Niikappu, and Harutachi from the west to east; S1 Table, Fig 1). Within the study region, several spawning rivers for *S*. *lanceolatus* were identified according to previous records [7, 8]. Sampling for eDNA was conducted from March to August in 2019, 14 times in total.

Seawater samples were collected at coastal sites with plastic dippers cleaned by soaking in a 10% sodium hypochlorite solution for 20 min before every use. Samples were moved into sterile plastic bags and 500 ml were immediately filtered using Sterivex^TM^-HV filter cartridges with a pore size of 0.45 μm (Merck Millipore, Inc., Darmstadt, Germany) and a sterile 50 ml syringe (TERUMO, Inc., Tokyo, Japan) following protocols outlined in the Environmental DNA Sampling and Experiment Manual version 2.1 by The eDNA Society (available from: http://ednasociety.org/eDNA_manual_Eng_v2_1_3b.pdf). Two filter cartridge samples were collected as field replicates per site, and negative control samples were prepared by filtration of 500 ml of ultrapure water at the end of every field sampling. All the filter samples were stored at -80°C until eDNA could be extracted.

### Environmental data collection

Four environmental factors; seawater temperature (°C), salinity (parts per thousand; ppt), daily mean Chlorophyll-a (Chl-a) concentration (mg/m^3^), and tidal height (cm) were collected. The seawater temperature and salinity were measured with a YSI Pro30 water quality sensor (Xylem, Inc., New York, USA) at each sampling site right after seawater sampling. The datasets of Chl-a concentration, an indicator of phytoplankton productivity, were collected from the P-Tree System database (https://www.eorc.jaxa.jp/ptree/index_j.html) supplied by the satellite Himawari-8, Japan Aerospace Exploration Agency (JAXA) [35, 36]. The daily mean values measured at the closest point to each eDNA sampling site were used. The tidal height datasets were collected from the database of oceanographic observatories maintained by the Japan Meteorological Agency (https://www.jma.go.jp/jma/indexe.html). The location of the station used is illustrated in Fig 1.

### DNA extraction and quantification

DNA extraction from the Sterivex^TM^-HV filter cartridge was performed largely following Yatsuyanagi et al. [8] with a slight modification of the vacuuming step. Firstly, RNAlater put into the filter cartridge was vacuumed from the outlet port using a QIAvac 24 Plus manifold (Qiagen, Inc., Hilden, Germany) and circulating aspirator (ADVANTEC, Inc., Tokyo, Japan). To remove the RNAlater, 1,000 μl ultrapure water was injected into the cartridges and vacuumed again (repeated twice). The eluted DNA solution was purified using a DNeasy Blood and Tissue Kit (Qiagen Inc.). The final volume of the extracted DNA was 100 μl.

DNA quantification was performed using a species-specific qPCR assay established in Yatsuyanagi et al. [8]. Details for PCR components, thermal cycling conditions, and standard curve preparation can also be found in Yatsuyanagi et al. [8]. In brief, quantitative PCR was run with a quantification standard in triplicate 20-μl reactions which included 800 nM of each primer, 400 nM probe, 0.1 μl Bovine Serum Albumin solution (20 mg/ml, New England BioLabs, Inc., Massachusetts, USA), and 2 μL of the DNA template in a Brilliant III Ultra-Fast qPCR Master Mix with Low ROX (Agilent Technology, Inc., California, USA). To test for cross-contamination, ultrapure water was applied as a PCR negative control, instead of the DNA template. In total, six replicates were analyzed per site, and the number of DNA copies per 2 μl template was calculated by averaging over them. The standard curve efficiency ranged from 0.910 to 1.000 and *R*^*2*^ ranged from 0.996 to 1.000 (intercept: 38.28–42.18). No DNA was detected in any of the negative controls. Based on the number of detected DNA copies, eDNA concentration per 1 ml of collected water was estimated following this formula:

$$\mathrm{eDNA}\;\mathrm{concentration}=\mathrm{DNA}\;\mathrm{number}\;(\mathrm{copies})\times \frac{\mathrm{DNA}\;\mathrm{extract}\;\mathrm{volume}\;(\mu l)}{\mathrm{DNA}\;\mathrm{template}\;\mathrm{volume}\;(\mu l)}\times \frac{1}{\mathrm{filtered}\;\mathrm{volume}\;(\mathrm{ml})}$$

which means that DNA copy number was divided by ten.

### Statistical analysis

To confirm the seasonal variability in temporal eDNA concentrations of *S*. *lanceolatus*, we compared the eDNA concentrations between spring (March-May, *n* = 49) and summer (June-August, *n* = 49) using Wilcoxon signed-rank test with R package “exactRankTests”.

We evaluated the relationships between eDNA concentrations and environmental factors using generalized linear mixed models (GLMMs) with negative binomial distribution. The eDNA concentration was used as a response variable, and the seawater temperature, salinity, Chl-a concentration, and tidal height datasets were used as explanatory variables. Sampling sites were treated as random effects. The GLMMs were conducted using the Automatic Differentiation Model Builder [37] with R package “glmmADMB”. Prior to GLMM analyses, we calculated variation inflation factors (VIFs) with R package “fmsb” to confirm the colinearity between the environmental factors. The VIFs of less than 3.000 indicate that the colinearity among the factors does not significantly influence the GLMMs [38]. All the statistical analyses were performed using R ver. 3-4-2 [39]. Heatmaps for data visualization were generated with HemI software [40].

## Results

### Spatial and temporal variation in eDNA concentrations

**Fig 2:**
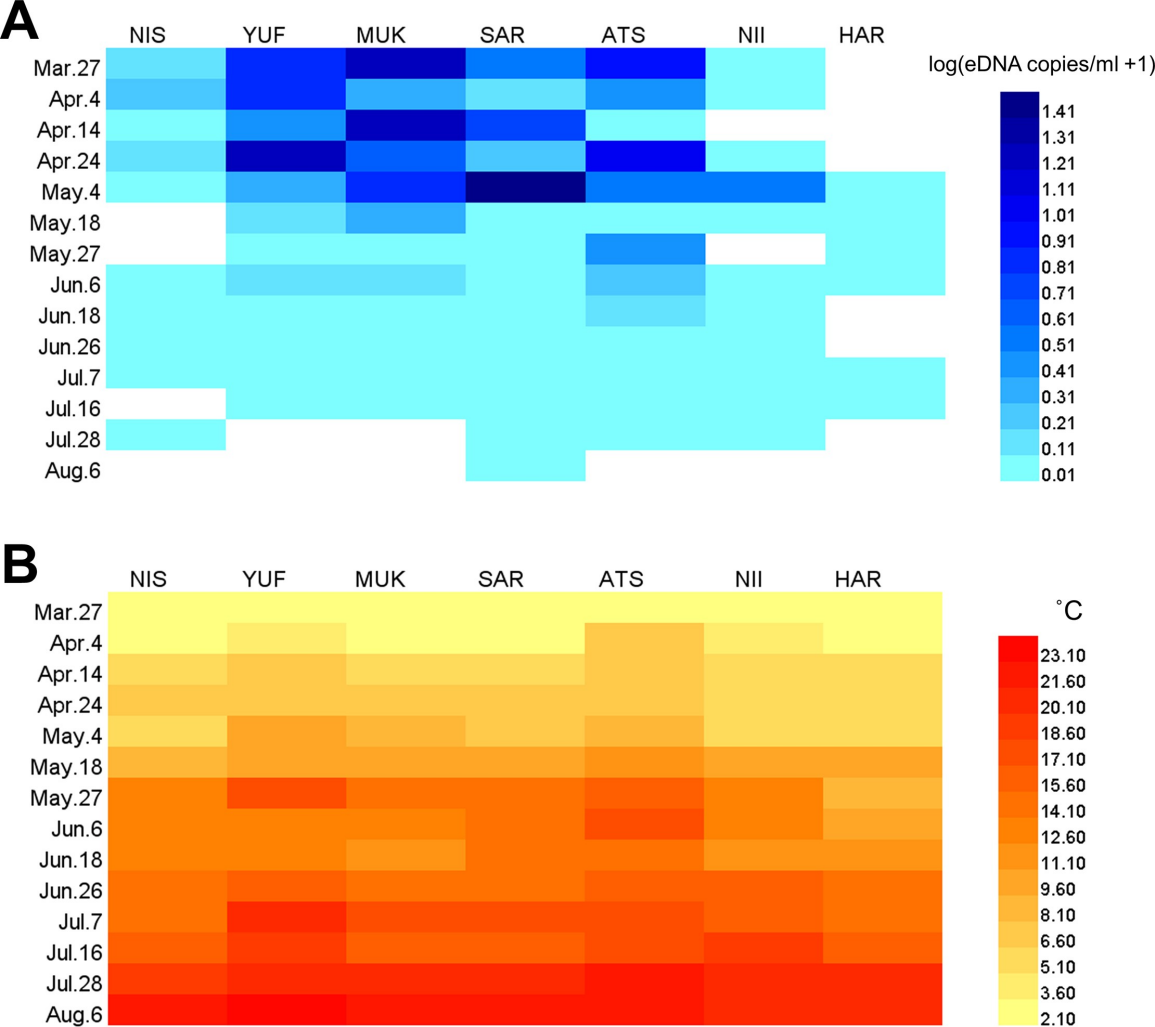
Heatmaps illustrating the spatiotemporal variation of A) eDNA concentrations and B) seawater temperature among sampling sites from March 27^th^ to August 6^th^, 2019. The x-axis and y-axis show the sampling sites and dates, respectively. Color gradations represents the log-transformed eDNA concentrations values [log_10_(eDNA copies/ml + 1)] and temperature (°C). Blank (white) columns represent no eDNA detection.

The eDNA shed by *S*. *lanceolatus* was detected in 73 out of the 98 seawater samples (Fig 2A). The minimum eDNA concentration (0.005 copies/ml) was obtained from Atsuga (ATS) on April 14^th^, and the maximum eDNA concentration (24.91 copies/ml) was from Saru (SAR) on May 4^th^. Of the 73 detections, 33 were below 1 copy per 2 μl of PCR template, which was categorized as below-the-detection level in S1 Fig (but not in Fig 2A). Given zero detections of *S*. *lanceolatus* eDNA from both field and PCR negative controls, those 33 samples were included in the following analyses as positive samples.

**Fig 3:**
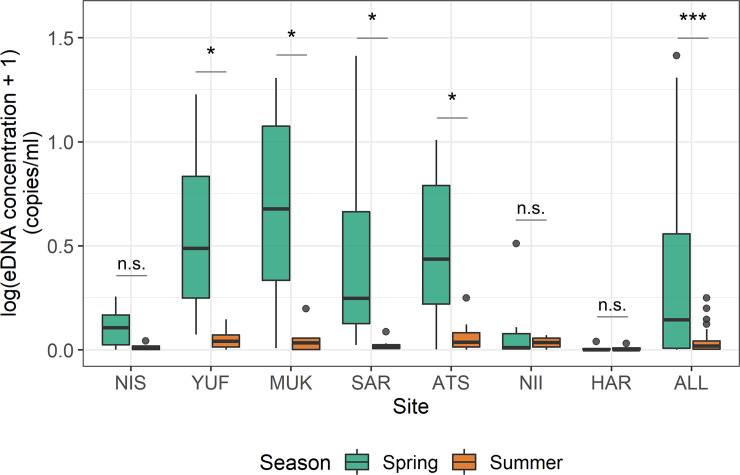
Boxplots of log-transformed eDNA concentrations among sites and comparisons between spring (March-May, *n* = 49) and summer (June-August, *n* = 49). Sites are Nishitappu (NIS), Yufutsu (YUF), Mukawa (MUK), Saru (SAR), Atsuga (ATS), Niikappu (NII), and Harutachi (HAR) and all pooling the data together (ALL). The horizontal lines in the boxplots represent the medians. “*”, “***”, and “n.s.” represent p < 0.05, p < 0.001, and non-significant difference, respectively.

In general, high eDNA concentrations were detected in the early sampling periods. In fact, the eDNA concentrations in Yufutsu (YUF), Mukawa (MUK), Saru (SAR), and Atsuga (ATS) showed significant differences between spring (March-May) and summer (June-August) (Wilcoxon signed-rank test; *P* < 0.05; Fig 3). Although results from the other sites (NIS, NII, and HAR) showed no significant differences in eDNA concentrations between the two seasons, an overall comparison pooling the data among sites suggested a significant difference between the seasons (*P* < 0.001, Fig 3).

**Fig 4:**
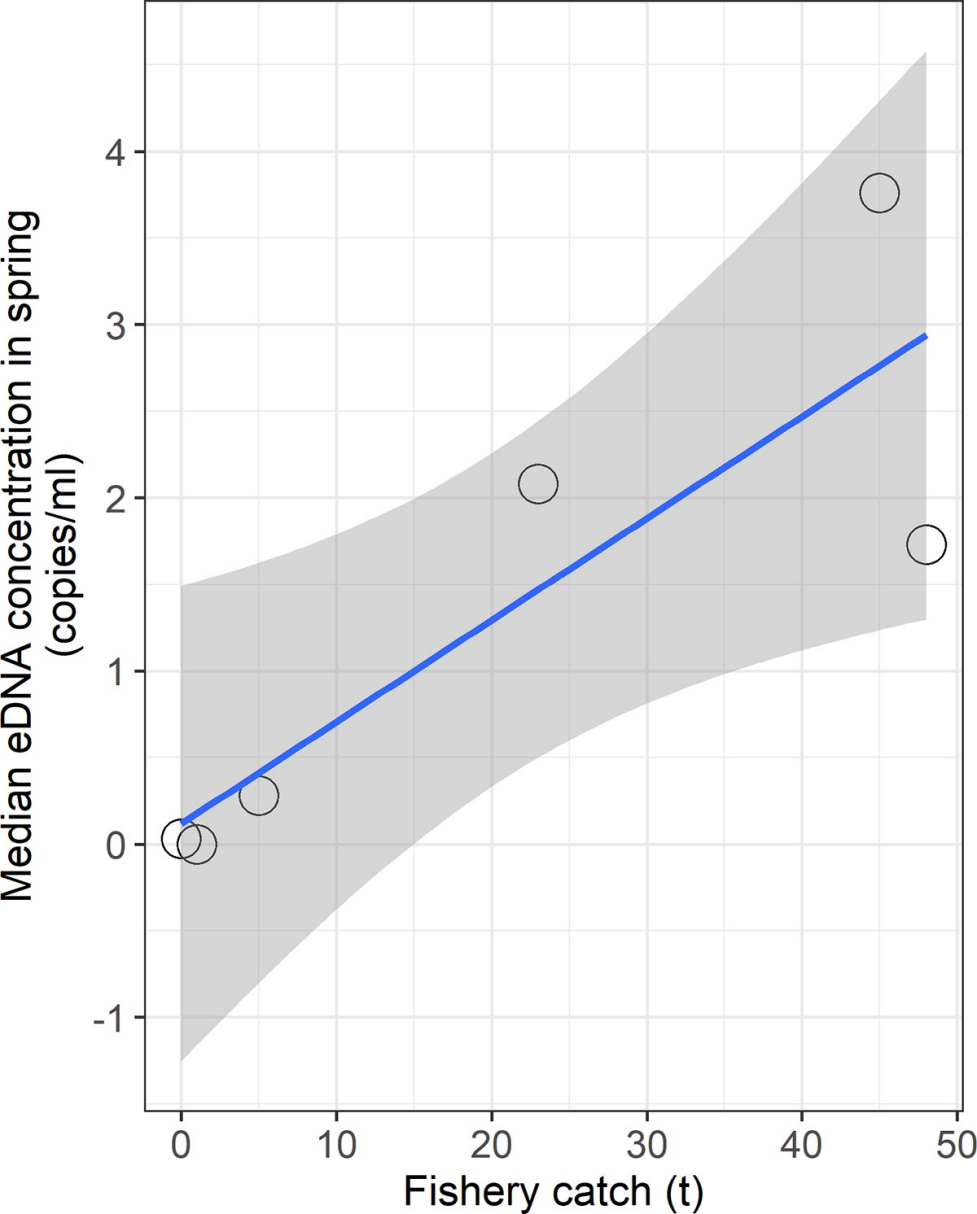
Relationship between eDNA concentration and regional fishery catch. The blue regression line represents a significantly positive correlation between median eDNA concentrations in seawater and fishery catch numbers in 2018 for six regions (*R*^*2*^ = 0.742, *P* < 0.05). Shaded area shows 95% confidence intervals for the regression model. The fishery catch data was provided by Hokkaido government under the CC BY 4.0 license [41].

According to catch reports filed by the Hokkaido Government [41], numbers of *S*. *lanceolatus* caught by fisheries between October and November in 2018 were larger in the central areas (around YUF: 23 tons, MUK: 45 tons, ATS: 48 tons) in comparison to the margins (around NIS: 5 tons, NII: less than 1 ton, HAR: 1 ton). The median eDNA concentrations detected in spring represented a significantly positive correlation with the regional fishery catches (*R*^*2*^ = 0.742, *P* < 0.05; Fig 4).

### Spatial and temporal variations in environmental factors

Variations in examined environmental factors are shown in Fig 2B (seawater temperature) and S2 Fig (others), and the VIFs among them are shown in S2 Table. The seawater temperature ranged from 2.1°C (in Mukawa on March 27^th^) to 23.2°C (in Yufutsu on August 6^th^). The salinity ranged from 19.8 ppt (in Yufutsu on July 7^th^) to 29.7 ppt (in Harutachi on April 4^th^). The Chl-a concentrations ranged from 35 g/m^3^ (in Saru on April 14^th^) to 1,275 g/m^3^ (in Nishikioka on June 26^th^). The tidal height ranged from 3 cm (in Atsuga at 12 noon on June 6^th^) to 129 cm (in Harutachi at 5 p.m. on June 18^th^). All the VIF values were less than 2, indicating that colinearity among the environmental factors did not significantly influence the GLMMs.

### Evaluating relationships between eDNA concentration and environmental factors

**Fig 5:**
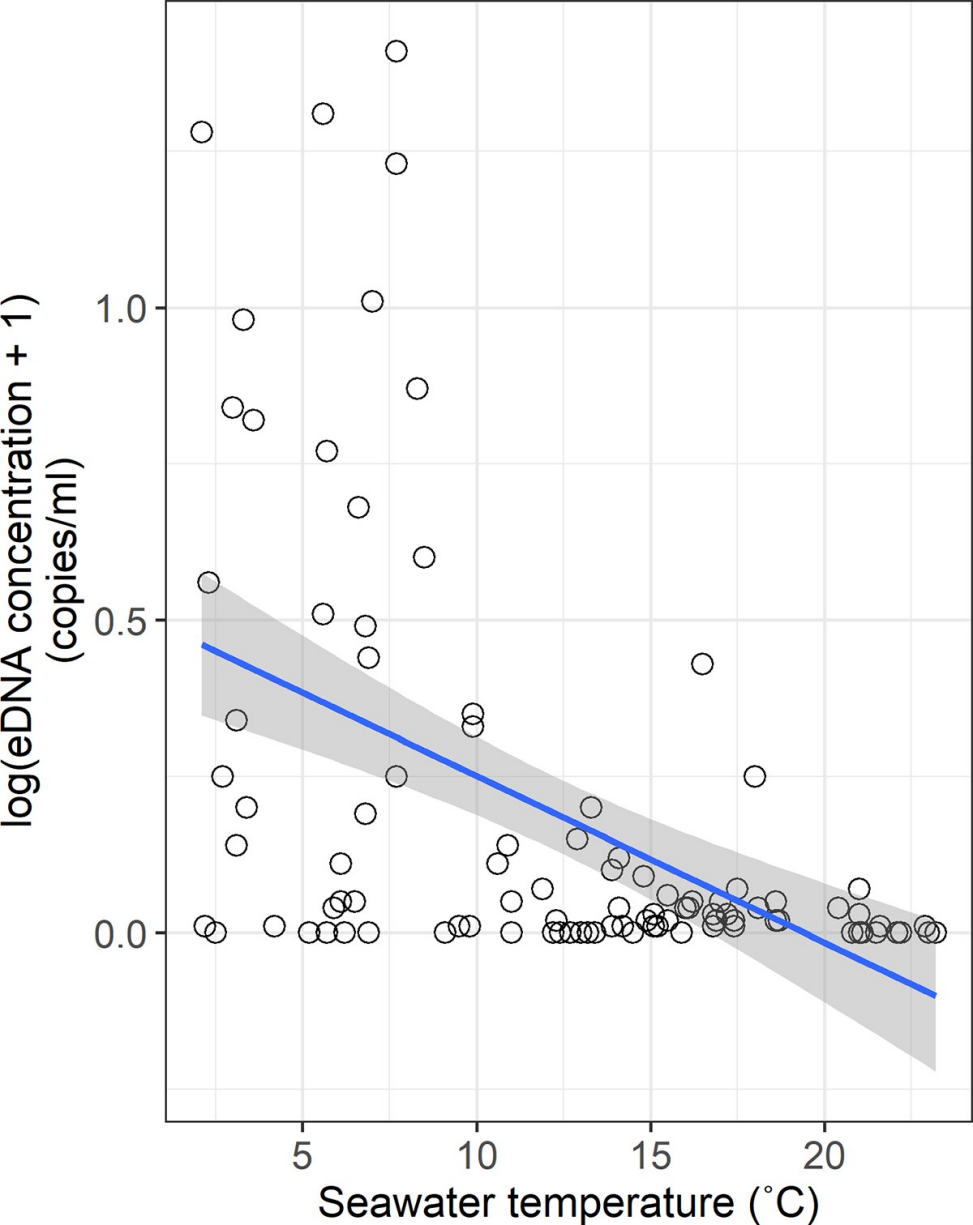
Relationship between log-transformed eDNA concentration and seawater temperature. The blue regression line represents a significantly negative correlation between them (*R*^*2*^ = 0.273, *P* < 0.001). Shaded area shows 95% confidence intervals.

GLMM and linear regression analyses suggested a significantly negative correlation between eDNA concentration and seawater temperature, indicating that eDNA concentrations declined gradually with increase of the seawater temperature (*R*^*2*^ = 0.273, *P* < 0.001; Table 1; Fig 5), whereas the salinity, Chl-a concentration, and tidal height were not significantly associated with the eDNA concentration.

**Table 1:** Summary of statistical analyses on GLMMs showing the variables and outputs.

Response variable	Explanatory variable	Estimate	*SD*	*z*-value	*P*-value
eDNA concentrations	Temperature	-2.75 x 10^−1^	3.82 x 10^−2^	-7.23	<2e-16
	Salinity	-1.68 x 10^−1^	9.85 x 10^−2^	-1.71	0.088
	Chl-a conc.	1.28 x 10^−3^	8.90 x 10^−4^	1.44	0.151
	Tidal height	5.51 x 10^−3^	7.62 x 10^−3^	0.72	0.470

The eDNA concentration was used as a response variable and the four environmental factors were used as the explanatory variables. Point estimates, standard deviations (*SD*s), *z*-values, and *P*-values were provided for each model.

## Discussion

In this study, a time-series eDNA survey for *S*. *lanceolatus* demonstrates the ability to trace the species’ coastal distribution and its spatiotemporal changes between spring and summer (Figs 2 and 3). Among eDNA detections, the highest eDNA concentration was detected in spring (March-May) around the central study sites, which was consistent with regional biomass inferred by fishery catch data from late fall (Fig 4). In summer (June-August), the eDNA concentrations decreased near shore, which, if the correlation between eDNA concentrations and fisheries catch data holds true, indicates *S*. *lanceolatus* habitat changes at that time. Currently, fish migration studies require a great amount of labor to monitor populations in a wide geographical range. Here, intensive eDNA sampling along a 100 km-long coastline enabled us to monitor the unseen migration trends of *S*. *lanceolatus*.

Indirect surveys using the eDNA approach do not provide detailed information (e.g., distance of sampling point to target organism) especially in open marine systems. However, recent eDNA studies have enhanced understandings about eDNA dispersion in coastal marine systems. Yamamoto et al. [25] compared eDNA concentrations with echo intensities in Japanese jack mackerel (*Trachurus japonicus*) and suggested that eDNA reflected relative abundance of those fish sources within 150 m in Maizuru Bay, the Sea of Japan. Using caged fishes in the Maizuru Bay, Murakami et al. [42] indicated that eDNA was detectable mostly within 30 m from the source. In the present study, *S*. *lanceolatus* likely stayed close to the shore in spring, at least around the central study sites according to the high eDNA concentrations detected.

The eDNA concentration of *S*. *lanceolatus* was significantly negatively correlated with the seawater temperature (GLMM, *P* < 0.001; Table 1; Fig 5). Previous studies reported that eDNA decays rapidly in warmer water [43–47]. For instance, Tsuji et al. [47] showed that eDNA in water at 25°C decayed 2.3 times faster than that at 15°C (−0.088/h at 15°C, −0.200/h at 25°C). Jo et al. [45] indicated that at higher water temperatures and with larger fish biomass, both eDNA shedding and eDNA decay rates increased. Among the study sites, however, the seawater temperature should not have been high enough to significantly decompose eDNA in June (11.0–16.8°C), suggesting that the decrease of eDNA concentrations was an ecological consequence of *S*. *lanceolatus* migration.

Water temperature in shallow coastal environments tends to be higher than that in deeper offshore spaces. In fact, temperatures at 50 m depth in the western Pacific Ocean off Hokkaido in June ranged from about 5.0°C to 7.0°C according to the Japan Meteorological Agency (http://www.jma.go.jp/jma/indexe.html). Together with the eDNA concentrations we estimated in the current study, we believe that *S*. *lanceolatus* migrates from nearshore/surface areas to relatively deeper layers in summer due to the increase in sea surface temperature. For a more precise understanding of habitat use in summer, however, further investigations with multi-depth water sampling will be needed.

The state of eDNA changes depending on various factors besides temperature, such as pH [48], ultraviolet radiation [49], biochemical oxygen demand, and Chl-a concentration [50]. Some previous studies suggested that tidal amplitude has little effect on temporal eDNA concentration in nearshore environment [42, 51]. In this study, tested environmental factors such as salinity, Chl-a concentration, and tidal height had little effect on the eDNA concentration of *S*. *lanceolatus* (Table 1). However, in order to interpret the eDNA data observed in open marine systems and improve the accuracy of biomass estimation, one needs to seek more detailed information on the relationships between eDNA dynamics and the environmental factors in seawater.

Smelt fishes, in the family Osmeridae, display diverse life-history characteristics with marine, anadromous, and freshwater forms. While the family has a widespread distribution, most Osmerid species have more limited ranges, generally along single coastlines of the North Pacific and North Atlantic. Notable exceptions include the Holarctic Osmerids, Capelin (*Mallotus villosus*) and Rainbow smelt (*Osmerus dentex*) [52]. Longfin smelt (*Spirinchus thaleichthys*) is closely related to *S*. *lanceolatus* and inhabits lakes, coastal river estuaries, and nearshore marine environments from Alaska to central California. *S*. *thaleichthys* juveniles aggregate in low-salinity estuaries from winter through spring and then outmigrate seaward during summer [53], showing the same seasonal movement pattern as *S*. *lanceolatus*. Our findings suggest the similarities in their phenology between the two closely related species for the first time.

This study uncovered new aspects of *S*. *lanceolatus* life history in the coastal regions. The outmigration of this species in summer reveals likely survival strategies for the species such as metabolic optimization or escape from predators. Osmerids are important forage fishes acting as energetic pathways between zooplankton and higher-trophic-level predators, which are keys for the maintenance of aquatic food webs and ecosystem functions [2, 54]. Therefore, continuous evaluation of the interaction between population dynamics of *S*. *lanceolatus* and environmental and community changes will be required for long-term management of this species and the surrounding ecosystems. Together with our previous eDNA study on the freshwater migration of *S*. *lanceolatus* [8], this study illustrates the usefulness of eDNA techniques for understanding the whole life history of this valuable endemic species.

## Supporting information

S1 Table Geographical information of seven eDNA sampling sites and sampling dates. (DOCX)

S2 Table Variance inflation factors (VIFs) among environmental factors. (DOCX)

S3 Table. All the raw data for quantitative PCR (XLSX)

S1 Fig Heatmap illustrating the spatiotemporal variation of eDNA concentrations (except for samples below 1 copy per 2 μl PCR template) among sampling sites from March 27^th^ to August 6^th^, 2019. (TIF)

S2 Fig Heatmaps illustrating the spatiotemporal variation of environmental factors. A) salinity (ppt), B) Chlorophyll-a concentration [log_10_(g/m^3^)], and C) tidal height (cm) among sampling sites from March 27^th^ to August 6^th^. Blank (white) columns in the heatmap of Chl-a concentrations mean that the data were not available due to weather disturbances.
(TIF)
