# Supplementary material for: Understanding seasonal migration of Shishamo smelt in coastal regions using environmental DNA
Source: PLoS One. 2020 Oct 1;15(10):e0239912. doi: 10.1371/journal.pone.0239912 (PMC7529200; doi:10.1371/journal.pone.0239912)
Supplement: S1 Table — (DOCX) [file pone.0239912.s001.docx]

**S1 Table. Geographical information of seven eDNA sampling sites and sampling dates**

| Location | Latitude/Longitude | Sampling date (2019) |
| --- | --- | --- |
| 1. Nishikioka (NIS) | 42.599010N/141.482650E | March 27^th^  April 4^th^, 14^th^, 24^th^  May 4^th^, 18^th^, 27^th^  June 6^th^, 18^th^, 26^th^  July 7^th^, 16^th^, 28^th^  August 6^th^ |
| 2. Yufutsu (YUF) | 42.621987N/141.756804E |  |
| 3. Mukawa (MUK) | 42.554670N/141.931290E |  |
| 4. Saru (SAR) | 42.487828N/142.025926E |  |
| 5. Atsuga (ATS) | 42.439451N/142.205603E |  |
| 6. Niikappu (NII) | 42.358100N/142.313010E |  |
| 7. Harutachi (HAR) | 42.266867N/142.497616E |  |
