# Supplementary material for: Understanding seasonal migration of Shishamo smelt in coastal regions using environmental DNA
Source: PLoS One. 2020 Oct 1;15(10):e0239912. doi: 10.1371/journal.pone.0239912 (PMC7529200; doi:10.1371/journal.pone.0239912)
Supplement: S2 Table — (DOCX) [file pone.0239912.s002.docx]

**S2 Table**: **Variance inflation factors (VIFs) among environmental factors**

|  | Salinity | Chl-a conc. | Tidal height |
| --- | --- | --- | --- |
| Temp. | 1.068 | 1.036 | 1.000 |
| Salinity |  | 1.079 | 1.000 |
| Chl-a conc. |  |  | 1.000 |
| Tidal height |  |  |  |

Each column shows VIF value calculated by one-to-one comparison between factors. The VIFs of less than 3.000 indicate that the colinearity among the factors does not significantly influence the GLMMs [37]. All the VIF values were less than 2, suggesting that there was no significant co-linearity among environmental factors.
