# Supplementary figures and images for: Understanding seasonal migration of Shishamo smelt in coastal regions using environmental DNA
Source: PLoS One. 2020 Oct 1;15(10):e0239912. doi: 10.1371/journal.pone.0239912 (PMC7529200; doi:10.1371/journal.pone.0239912)

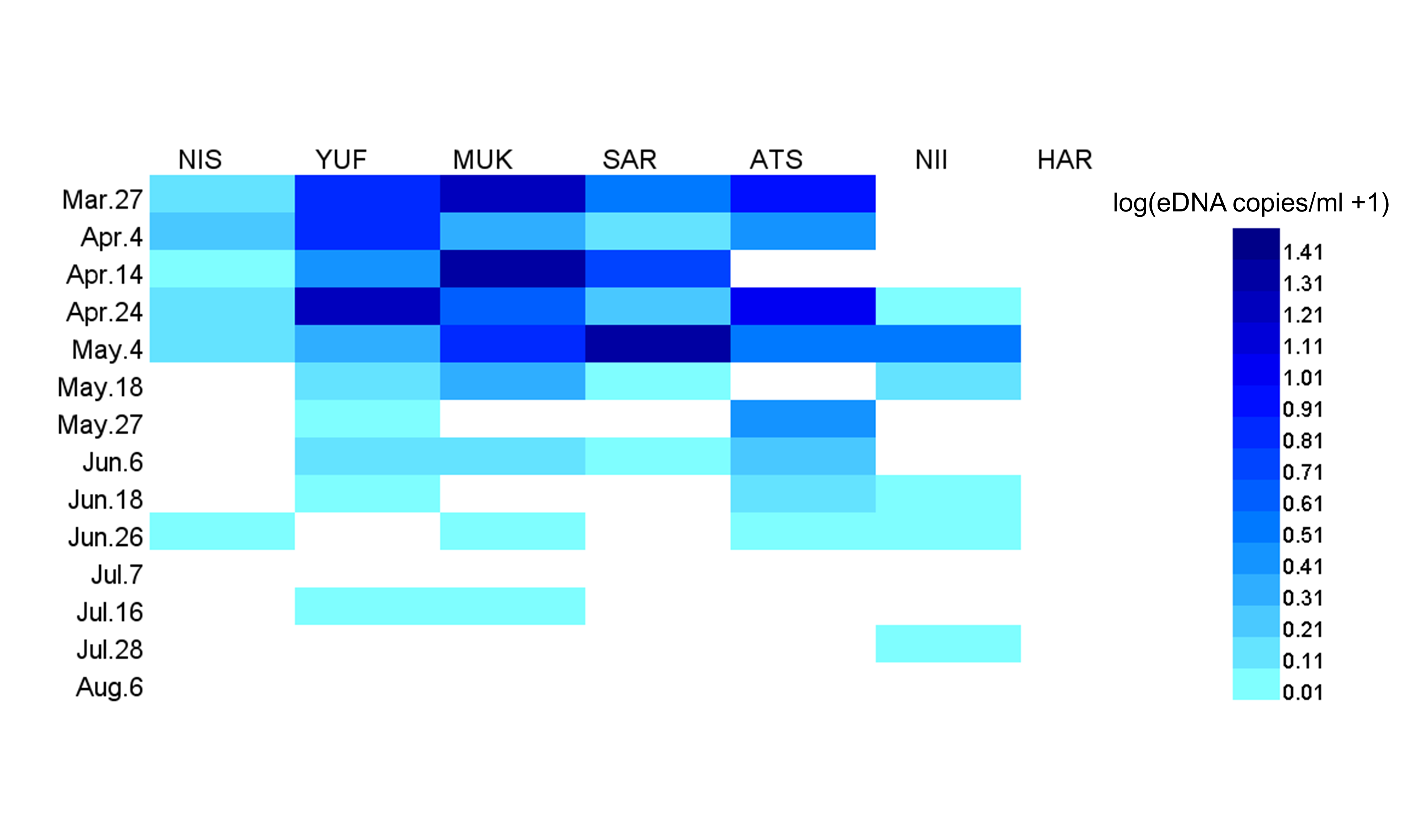

Supplement: S1 Fig — (TIF) [file pone.0239912.s004.tif]

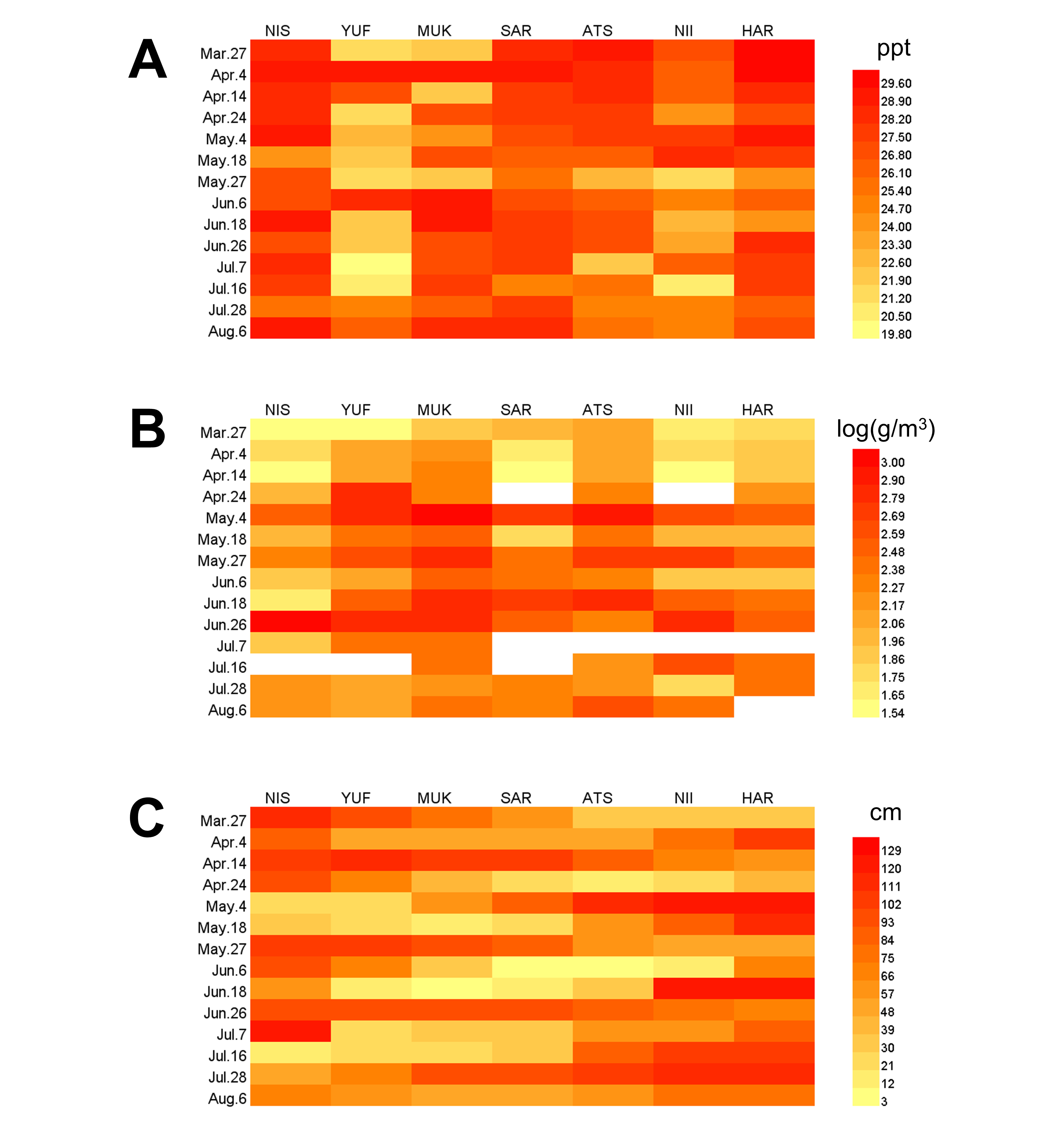

Supplement: S2 Fig — A) salinity (ppt), B) Chlorophyll-a concentration [log10(g/m3)], and C) tidal height (cm) among sampling sites from March 27th to August 6th. Blank (white) columns in the heatmap of Chl-a concentrations mean that the data were not available due to weather disturbances. (TIF) [file pone.0239912.s005.tif]
